# Total gastric necrosis following acute pancreatitis in a patient with COVID -19: Case report and literature review

**DOI:** 10.1016/j.amsu.2021.01.061

**Published:** 2021-01-21

**Authors:** Mounir Bouali, Mohamed Ouchane, Abdelilah Elbakouri, Fatimazahra Bensardi, Khalid Elhattabi, Abdelaziz Fadil

**Affiliations:** Visceral Surgical Emergency Department, Faculty of Medicine and Pharmacy, Universitary Hospital Center Ibn Rochd, Hassan II University, Casablanca, Morocco

**Keywords:** COVID −19, Acute pancreatitis, Gastric necrosis, Surgery, Case report

## Abstract

The Coronavirus Disease Pandemic - 2019 (COVID-19) has spread to more than 200 countries worldwide, affecting more than 2 million people and >120,000 deaths, Acute pancreatitis of infectious origin can be caused by different viruses but currently no study has concluded that COVID-19 is directly responsible for acute pancreatitis. We report the case of a COVID-19 patient admitted to the emergency room in a state of respiratory distress associated with stage E pancreatitis according to the classification of balthazar whose surgical exploration found total necrosis of the stomach. A total gastrectomy was performed with an esophagostomy and a wide drainage of the abdominal cavity, the postoperative sequelae were marked by the death of the patient at D6 postoperatively by cardiorespiratory arrest.

## Highlights

• The Coronavirus Disease Pandemic - 2019 (COVID-19) has spread to more than 200 countries worldwide, affecting more than 2 million people and >120,000 deaths.
• Acute pancreatitis of infectious origin can be caused by different viruses but currently no study has concluded that COVID-19 is directly responsible for acute pancreatitis.
• We report the case of a COVID-19 patient with stage E pancreatitis whose surgical exploration found total necrosis of the stomach.

## 1 Introduction

The Coronavirus Disease Pandemic - 2019 (COVID-19) has spread to more than 200 countries worldwide, affecting more than 2 million people and >120,000 deaths. The COVID-19 disease mainly affects the respiratory tract, digestive symptoms are often associated with abdominal pain, vomiting, diarrhea …. Acute pancreatitis of infectious origin can be caused by different viruses such as mumps, measles, coxsackie, Epstein-Barr virus, hepatitis A virus, human immunodeficiency virus, cytomegalovirus and influenza A (H1N1) but currently no study has concluded that COVID-19 is directly responsible for acute pancreatitis. We report the case of a COVID-19 patient admitted to the emergency room in a state of respiratory distress associated with stage E pancreatitis according to the balthazar classification, whose surgical exploration found total necrosis of the stomach.this article is respecting the SCARE Checklist guidelines [1].

## 2 Patient and observation

**Fig. 1:**
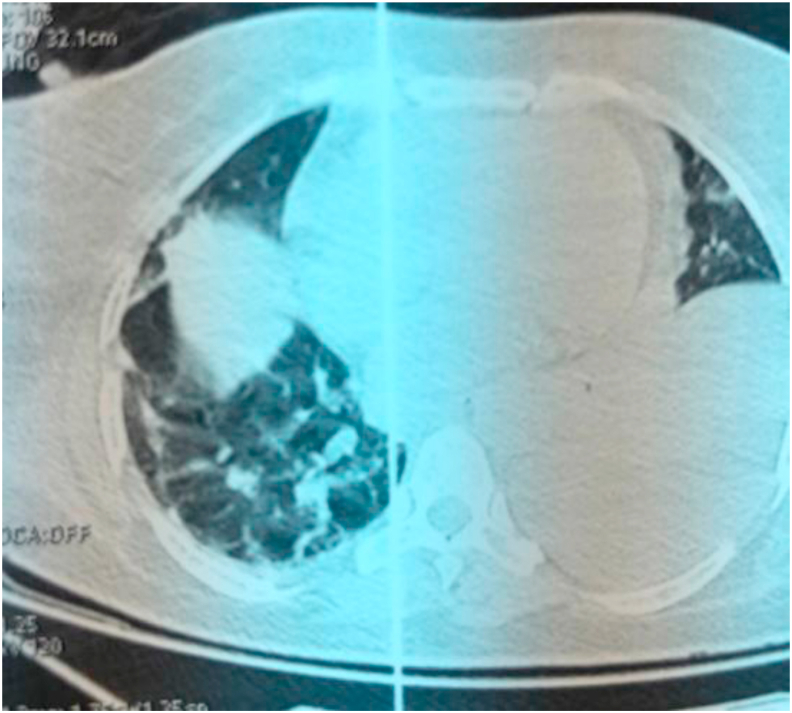
CT image showing multiple round and oval ground-glass opacities in both lungs, with a crazy-paving pattern in relation to COVID-19.

**Fig. 2:**
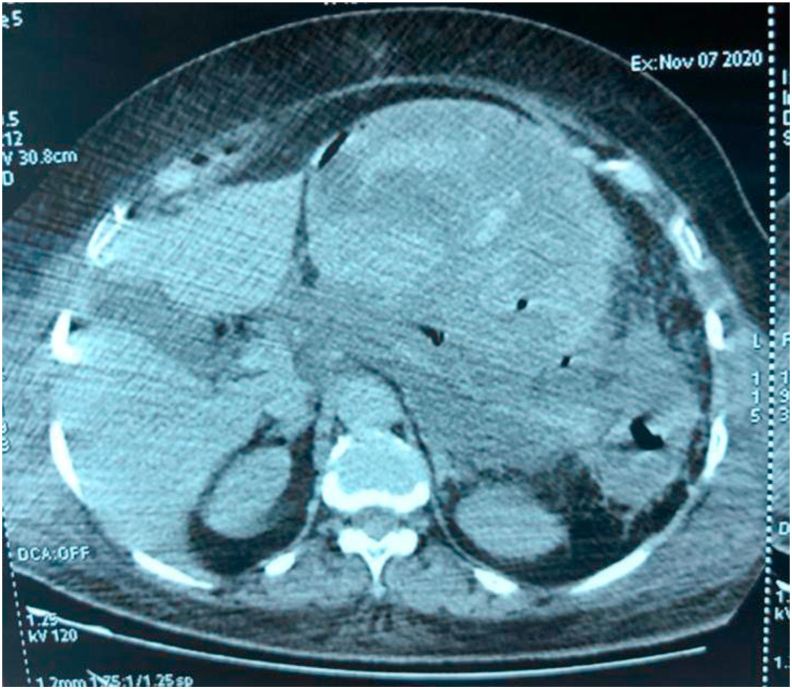
CT appearance of stage E pancreatitis according to the Balthazar classification with peripancreatic necrotic collections.

This is a 60-year-old female patient with a BMI of 24 kg/m2 without any particular pathological history admitted to the surgical emergency room in a respiratory distress with diffuse abdominal pain associated with hematemesis and melaenas and in whom the clinical examination found a conscious patient, BP: 09/04 mmhg, HR: 109 bpm, T°: 39, respiratory rate: 24 cycles per minute, free oxygen saturation 89%, the abdominal examination finds generalized abdominal tenderness. The abdominal thoraco abdominal CT scan had shown multiple round and oval ground-glass opacities in both lungs, with a crazy-paving pattern (Fig. 1), on the abdominal pancreatitis stage E associated with superinfected peripancreatic collections (Fig. 2).

**Fig. 3:**
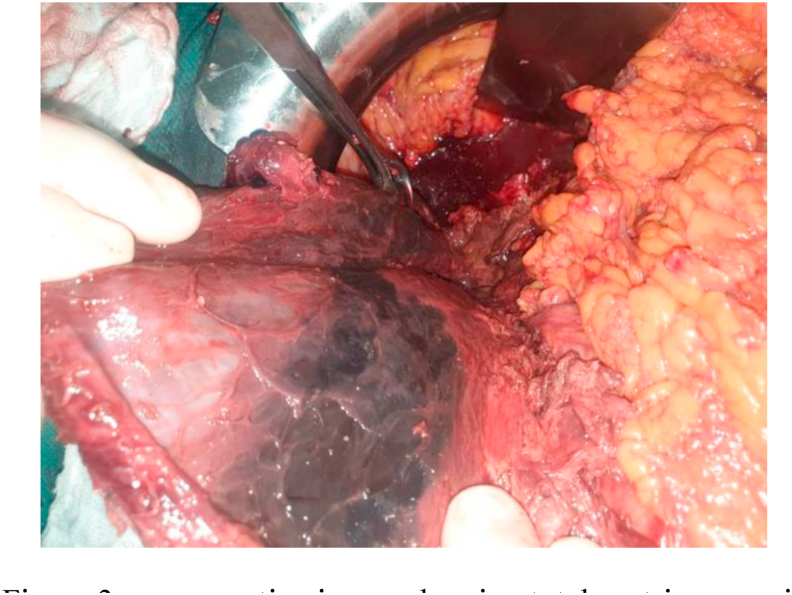
Per operative image showing total gastric necrosis.

The biological checkup showed hyperleukocytosis at 23,0000/mm3, anemia with hemoglobin 9g/dl and platelet count at 150,000/mm3, Lipase 627 U/liter, COVID-19 PCR was positive, Procalcitonin 30 ng/ml and C-reactive protein at 80 mg/liter. After monitoring and conditioning, surgical exploration by median laparotomy showed total gastric necrosis (Fig. 3) with necrosis flows in the back cavity of the epiploons.

A total gastrectomy was performed with an esophagostomy and a wide drainage of the abdominal cavity, the postoperative sequelae were marked by the death of the patient at D6 postoperatively by cardiorespiratory arrest.

## 3 Discussion

Acute pancreatitis is an acute inflammation of the pancreas with an estimated incidence of 30 per 100,000 in men and 20 per 100,000 in women (1) There are two forms: benign acute pancreatitis, known as edematous, and acute necrotizing pancreatitis. The incidence of acute pancreatitis of viral origin is so far unknown [2] The viruses that are known to cause pancreatitis are mumps, measles, coxsackie, Epstein-Barr virus, hepatitis A virus, human immunodeficiency virus, cytomegalovirus and influenza A (H1N1). The pandemic situation of the coronavirus disease - 2019 (COVID-19) leads us to look for a relationship between COVID-19 and acute pancreatitis since abdominal pain is part of the symptoms of both diseases. In a retrospective cohort study analyzing 11,883 patients hospitalized with COVID-19 in 12 hospitals in the United States, there were 32 cases of acute pancreatitis, giving a prevalence of 0.27%, of which 69% are idiopathic [3] Severe Acute Respiratory Syndrome Coronavirus 2 (SARS-CoV-2), the causative agent of COVID-19, uses Angiotensin converting enzyme 2 (ACE2) to enter human cells and TMPRSS2 to “prime" [4]. Both proteins are expressed in epithelial cells of the gastrointestinal tract and the virus is isolated in the feces. Pancreatic duct cells, acinar cells and pancreatic islet cells also express ACE2, making infection of the gland plausible as the virus spreads from duodenal epithelium to the pancreatic duct and then to acinar and islet cells [5]. SARS CoV-2 has been isolated from samples from a pancreatic pseudocyst from a patient with acute pancreatitis [6]. SARS CoV-2 is particularly capable of causing severe diffuse endothelitis of submucosal vessels in several anatomical sites, and these changes that cause diffuse ischemia of certain organs [7] but to date the direct association between COVID-19 and acute pancreatitis is not based on evidence. During acute pancreatitis the pancreas will undergo alterations in its microcirculation in the form of vasoconstriction and progressive exclusion of capillaries resulting in a decrease in local vascularization promoting the appearance of necrosis which may be systemic due to the significant release of inflammatory mediators such as IL-1A and phospholipase A2 which induce global endothelial dysfunction and increase vascular permeability [9]. Necrosis of the stomach is rare and usually due to the presence of thrombosis and stenosis of gastric vessels [10]. During acute necrotizing pancreatitis, Acute Necrotic Collections contain varying amounts of fluid and necrotic debris from the pancreatic parenchyma [11]. Thoraco-abdominal CT scan should be requested in this pandemic context for signs related to COVID-19 and for signs of severity and complications of acute pancreatitis. Surgical management in infected necrotic casts is an open necrosectomy to remove infected necrotic tissue. Several drains are left in place to allow continuous irrigation and lavage of the abdominal cavity; The mortality rate is more than 50% in case of early surgery (<72 hours), Trans gastric drainage by endoscopy is an efficient alternative but requires a multidisciplinary consultation [11], in our case the surgical approach was necessary due to gastric necrosis and hemodynamic and respiratory instability. This case should teach us to check the Lipase in front of every abdominal pain in situation of COVID-19 and check the pancreas state by an abdominal CT scan.

## 4 Conclusion

No scientific evidence has confirmed the direct association between COVID-19 and acute pancreatitis, hence the importance of measuring lipase in patients with SARS CoV-2 infection, particularly in cases of acute abdominal pain, our case was a woman with COVID-19 admitted for stage E pancreatitis with intraoperative discovery of total gastric necrosis.

## Ethical approval

I declare on my honor that the ethical approval has been exempted by my establishment.

## Sources of funding

None.

## Author contribution

Bouali Mounir: writing the paper.

El Hattabi Khalid: study concept.

Ouchane mohamed: Corresponding author writing the paper and operating surgeon.

El Bakouri Abdelilah: writing the paper and operating surgeon.

El attar Layla: writing the paper.

Bensardi Fatimazahra: study concept.

Fadil Abdelaziz: correction of the paper.

## Consent

Written informed consent was obtained from the patient for publication of this case report and accompanying images. A copy of the written consent is available for review by the Editor-in-Chief of this journal on request.

## Registration of research studies

Researchregistry2464.

## Guarantor

Pr Mounir bouali.

## Provenance and peer review

Not commissioned, externally peer reviewed.

## Declaration of competing interest

Authors have declared that no competing interests exist.
